# New insight into the mechanism of mitochondrial cytochrome c function

**DOI:** 10.1371/journal.pone.0178280

**Published:** 2017-05-31

**Authors:** Rita V. Chertkova, Nadezda A. Brazhe, Tatiana V. Bryantseva, Alexey N. Nekrasov, Dmitry A. Dolgikh, Alexander I. Yusipovich, Olga Sosnovtseva, Georgy V. Maksimov, Andrei B. Rubin, Mikhail P. Kirpichnikov

**Affiliations:** 1 Shemyakin-Ovchinnikov Institute of Bioorganic Chemistry, The Russian Academy of Sciences, Moscow, Russia; 2 Biophysics Department, Biological faculty, M.V. Lomonosov Moscow State University, Moscow, Russia; 3 Department of Biomedical Sciences, Faculty of Health and Medical Sciences, Copenhagen University, Copenhagen, Denmark; Russian Academy of Medical Sciences, RUSSIAN FEDERATION

## Abstract

We investigate functional role of the P^76^GTKMIFA^83^ fragment of the primary structure of cytochrome *c*. Based on the data obtained by the analysis of informational structure (ANIS), we propose a model of functioning of cytochrome *c*. According to this model, conformational rearrangements of the P^76^GTKMIFA^83^ loop fragment have a significant effect on conformational mobility of the heme. It is suggested that the conformational mobility of cytochrome *c* heme is responsible for its optimal orientation with respect to electron donor and acceptor within ubiquinol–cytochrome *c* oxidoreductase (complex III) and cytochrome *c* oxidase (complex IV), respectively, thus, ensuring electron transfer from complex III to complex IV. To validate the model, we design several mutant variants of horse cytochrome *c* with multiple substitutions of amino acid residues in the P^76^GTKMIFA^83^ sequence that reduce its ability to undergo conformational rearrangements. With this, we study the succinate–cytochrome *c* reductase and cytochrome *c* oxidase activities of rat liver mitoplasts in the presence of mutant variants of cytochrome *c*. The electron transport activity of the mutant variants decreases to different extent. Resonance Raman spectroscopy (RRS) and surface-enhanced Raman spectroscopy (SERS) data demonstrate, that all mutant cytochromes possess heme with the higher degree of ruffling deformation, than that of the wild-type (WT) cytochrome *c*. The increase in the ruffled deformation of the heme of oxidized cytochromes correlated with the decrease in the electron transport rate of ubiquinol–cytochrome *c* reductase (complex III). Besides, all mutant cytochromes have lower mobility of the pyrrol rings and methine bridges, than WT cytochrome *c*. We show that a decrease in electron transport activity in the mutant variants correlates with conformational changes and reduced mobility of heme porphyrin. This points to a significant role of the P^76^GTKMIFA^83^ fragment in the electron transport function of cytochrome *c*.

## Introduction

Cytochrome *c* is a small globular protein containing iron porphyrin cofactor (heme *c*) that is covalently bound to the only polypeptide chain. The main function of cytochrome *c* is its involvement in the electron transport chain of the mitochondrial inner membrane. It is a key element that ensures cellular respiration. As an electron is transferred from ubiquinol–cytochrome *c* reductase (complex III) to cytochrome *c* oxidase (complex IV) in the mitochondrial respiratory chain, cytochrome *c* is reversibly reduced and oxidized. Short-living complexes of cytochrome *c* with its redox partner proteins must be formed for an electron to be transferred. According to the modern concept, the universal site of interaction between cytochrome *c* and complexes III and IV consists of the central hydrophobic domain and the surrounding electrostatic domain [1, 2]. Long-range electrostatic interactions determine correct spatial orientation of the contacting proteins, while hydrophobic interactions are often considered as the main force stabilizing the protein–protein complex. Electrostatic interactions are formed between the cluster of positively charged Lys residues on the cytochrome *c* surface around the heme cleft and the negatively charged amino acid residues residing on subunits of redox partner proteins that interact with cytochrome *c* [3, 4]. It is known that among the existing 19 Lys residues in equine cytochrome *c*, the conserved Lys residues at the positions 8, 13, 72, 73, 86, and 87 make the main contribution to electrostatic interaction, while Lys residues at the positions 5, 7, 25, 27, 79, and 88 occupy the periphery of the contact surface and are involved in binding to a lesser extent [3–6]. Cytochrome *c* residues Gln12, Lys13, Gln16, Lys27, Thr28, Ile81, and Ala83 are particularly important for stabilizing the protein electron transport complex. These residues are recruited in stacking and hydrophobic interactions as well as in van der Waals contacts with amino acid residues of the subunits of its redox partners [7–9].

On the basis of studies of the contribution of individual Lys residues of horse cytochrome *c* to the formation of reactive complexes with partner proteins [10], we designed a few mutant variants of cytochrome *c* with various combinations of substitutions of the positively charged Lys residues at the positions 8, 27, 72, 86, and 87 for the negatively charged Glu residues. All resulting mutants are characterized by reduced electron transport activity. One of them was successfully used as a basis for quantification of superoxide anion radical [11]. The decrease in electron transport activity was caused by elimination of electrostatic interactions between cytochrome *c* and partner proteins. Due to this, the mutant variants either became unable to form reactive complexes or had an orientation unfavorable for electron transport. However, a significant number of mutations substantially changed the total charge of the protein molecule to achieve this. In this study, we assumed that the electron transport activity of cytochrome *c* can be controlled by the labile loop containing the Met80 amino acid residue that coordinates the iron atom and has a positive effect on conformational mobility of cytochrome *c* heme.

In our work, analysis of the structure of cytochrome *c* molecule and construction of mutant protein variants were carried out by ANalysis of the Information Structure of protein (ANIS) method [12]. This method is an effective tool for the design of active forms of enzymes or chimeric proteins that combine the enzymatic activities of their wild-type prototypes. This method was successfully used to construct the active forms of certain proteins, e.g, human 1-CYS peroxiredoxin [13] and interleukin-13 antagonist [14]. The method allows for identification of protein structure elements responsible for catalytic activity. ANIS is based on the use of the primary protein amino acid sequence to reveal a hierarchy of the ELements of Information Structure (ELIS). ELIS corresponds to the variable length sites with an increased density of structural information. The amino acid residues forming the enzyme catalytic site were shown to belong to different top-ranking ELIS located in the contact area of the corresponding spatial structure clusters. In the protein, spatial structure catalytic sites are located in the area of contact between fragments of polypeptide chain (structural blocks) allocation to the different top-ranking ELIS [15].

The paradigm of “determinate mobility” of structural elements of proteins corresponding to ELIS was proposed to explain the mechanism of functioning of hydrolytic enzymes [15]. The “determinate mobility” causes changes in catalytic sites that ensure enzymatic reactions. In this study, we adopt this paradigm to explain mechanisms of functioning of heme-containing proteins.

Literature data suggest that the mechanism of changes in heme conformation and out-of-plane displacement of the Fe atom, which is observed for the hemoglobin β-subunit, may be applied for heme-containing proteins [16, 17]. Recently, we studied informational structure of cytochrome *c* [18] and compared it to the hemoglobin β-subunit using the ANIS method. In both proteins, amino acid residues interacting with the Fe atom in heme reside on different ELIS. Each of these residues (His87 for the β-chain of hemoglobin and Met80 for cytochrome *c*) is located at the only site with the abnormally low density of bottom-ranking elements in the informational structure of proteins (the ADD- site) [18, 19]. These sites are characterized by increased ability to change their conformation (flexibility), due to which the determinate mobility of ELIS is implemented according to the paradigm [20]. The revealed similarity between the informational structures of the β-subunit of hemoglobin and cytochrome *c* [19] may also point to similarity between the mechanisms of functioning of these proteins. We suggested that conformational rearrangements in the ADD- site of cytochrome *c* give rise to forces that alter the conformation of the entire heme, which may be accompanied by out-of-plane displacement of the Fe atom from the heme.

In this paper, we investigated the structural and the functional role of the only ADD- site in the polypeptide chain of horse cytochrome *c*, which corresponds to the P^76^GTKMIFA^83^ polypeptide sequence, in implementation of the electron transport. This region was selected as a target for directed mutagenesis since it was proposed as a key site of functioning of heme proteins [19]. We focused on how the introduction of amino acid substitutions to the P^76^GTKMIFA^83^ sequence that increase the rigidity of this domain affects heme conformation and functional activity of cytochrome *c*.

## Materials and methods

Material used in this studies were: components for the culture media and buffer solutions for chromatography and electrophoresis (AppliChem, Germany), ampicillin, cytochrome *c* from equine heart (Sigma, United States), *Xho* I restriction endonuclease (Promega, USA), *Bam*H I restriction endonuclease (New England Biolabs Inc., USA), *Pfu*-DNA polymerase, and T4-DNA ligase (Fermentas, Lithuania). Distilled water was additionally purified on a Milli-Q system (Millipore, USA).

### ANIS method

The informational structure of cytochrome *c* was calculated according to the algorithm described in details in Ref. [21]. The problems of structural and functional ELIS-first ranking role were discussed in Ref. [20].

### Construction of the mutant genes of cytochrome *c*

The mutations were introduced into the gene of horse cytochrome *c* in a composition with pBP(CYCS) expression plasmid vector by site-specific mutagenesis according to the QuikChangeTM Mutagenesis Kit method (Stratagene, USA) [22]. The cytochrome *c* genes with mutations in the (76–83) region were prepared using oligonucleotide primers with the corresponding substitutions (Table 1). The reaction mixture (50 μl) contained 10–15 ng of matrix DNA (a pBP(CYCS) plasmid containing the horse cytochrome *c* gene), oligonucleotide primers (125 ng), four deoxynucleoside triphosphates (10 nmol of each), and *Pfu* polymerase (2.5 activity units). Twenty cycles of the amplification reaction were performed according to the following scheme: denaturation of the matrix DNA at 95°C for 45 s, annealing at the calculated temperature for 60 s, and elongation at 74°C for 10 min. When the reaction was completed, the *Dpn* I restrictase (10 activity units) was added and the reaction mixture was incubated for 60 min at 37°C. Further, aliquots of the prepared mixture were used for transformation of the XL-1 Blue supercompetent cells of *E*. *coli* according to the standard procedure. The production of mutant DNA during mutagenesis was analyzed by electrophoresis in 1% agarose gel. The selected mutant genes were cloned in the pBP(CYC1) expression vector [23] modified for the expression of genes of horse cytochrome *c* [24]. The nucleotide sequences of mutant genes in the plasmid DNA were determined on an ABI Prism 3100-Avant Genetic Analyzer (Applied Biosystems, USA).

**Table 1:** The structure of the oligonucleotides (direct primers) used to introduce mutations into a sequence of horse cytochrome *c*.

Primer	Oligonucleotides structure 5'-3'
**T89A-dir**	GTATTAAGAAGAAGGCTGAAAGAGAAG
**T78S/K79P-dir**	GAAGTACATTCCTGGTAGCCCGATGATTTTCGCTGG
**I75G/G77R/T78I-dir**	CCCAAAGAAGTACGGTCCTCGTATCAAGATGATTTTCGC
**I81Y/A83Y/G84N-dir**	CATTCCTGGTACTAAGATGTATTTCTACAATATTAAGAAGAAGACTGAAAG
**K79V/I81L/F82R-dir**	GTACATTCCTGGTACTGTGATGCTGCGCGCTGGTATTAAGAAG
**I81L/F82S/A83S/G84A-dir**	CTGGTACTAAGATGCTGTCCTCTGCTATTAAGAAGAAGAC
**T78A/K79A/I81A/F82T-dir**	GTACATTCCTGGTGCTGCGATGGCTACCGCTGGTATTAAG
**T78N/K79Y/M80I/I81M/F82N-dir**	CAAAGAAGTACATTCCTGGTAACTACATCATGAACGCTGGTATTAAGAAGAAGAC

### Expression of the mutant genes of cytochrome *c*

**Expression of the mutant genes of cytochrome *c*** was performed in the JM-109 strain of *E*.*coli* in an SB liquid-nutrient medium with ampicillin (the final concentration was 200 μg/ml) without addition of the inductor at 37°C under vigorous stirring for 22–24 h [24].

After the growth of *E*.*coli* cells had been finished, they were precipitated by centrifugation at 4000 g and 4°C for 20 min. The cellular precipitate was resuspended in a buffer (25 mM NaP_i_, pH 6.0, 1 mM NaN_3_) and frozen at -20°C for 20–30 min. The cells were homogenized by forcing through a French press (Spectronic Instruments, Inc., USA) at high pressure with subsequent centrifugation at 100 000g for 20 min.

### Isolation and purification of cytochrome *c*

**Isolation and purification of the target proteins** were performed on a BioLogic HR liquid chromatographic system (Bio-Rad, USA) according to the previously elaborated scheme [25, 26]. The cellular extract was applied to a Mono S HR 10/10 cation–exchange column (Bio-Rad, USA), which was equilibrated with a buffer containing 25mM NaP_i_ (pH 6.0) and 1 mM NaN_3_. Cytochrome *c* was eluted with linear gradient of 1 M NaCl in the same buffer at a flow rate of 3 ml/min. The fraction obtained after purification on the Mono S column was analyzed on a spectrophotometer and by SDS-PAAG electrophoresis, dialyzed against the buffer for absorption chromatography (10 mM NaP_i_, pH 7.0, 1 mM NaN_3_), and applied to a column with CHT-I hydroxyapatite (Bio-Rad, USA). Cytochrome *c* was eluted with a linear gradient of 500 mM NaP_i_ with pH 7.0 at a flow rate of 1 ml/min. The degree of purification and concentration of cytochrome *c* in the resulting fractions were determined on a spectrophotometer and by SDS-PAAG electrophoresis. The fractions with the A_409_/A_280_ purity of 4.5–5.0 (this value corresponded to purity of ≥95% for the substance commercially prepared by Sigma, USA) were oxidized by treating with potassium ferricyanide added at the equimolar concentration, dialyzed three times against 10 mM ammonium carbonate buffer (pH 7.9), and lyophilized on an ALPHA I-5 device. Absorption spectra of the cytochrome samples after the oxidation with potassium ferricyanide, after the two-fold dialysis for 20 hours and after the lyophilisation and dissolving in 10 mM phosphate buffer were the same and resemble fully oxidized cytochrome *c* (Amax 409 nm, one peak in the region of 530–540 nm that is typical for fully oxidized cytochrome *c*). To verify the absence of the reduced form of cytochrome in the sample, recordings of the absorption spectra were carried out right before the studies of the biological activity of cytochromes in preparations of SMP and before SERS measurements.

### Preparation of mitoplasts without cytochrome *c*

Mitochondria from rat liver were prepared using the Johnson-Lardy method [27]. Mitoplasts (mitochondria lacking the outer membrane and cytochrome *c*) were prepared according to the Jacobs-Sanadi method [28]. Three month-old male Wistar rats born in the vivarium of the Faculty of Biology, M.V. Lomonosov Moscow State University were used. The animals were housed at 22°C in light-controlled environment (12:12 h light-dark cycle) and had free access to water and food. Living conditions, all procedures involving animals and the protocol of experiments were approved by Bioethics Committee of M.V. Lomonosov Moscow State University according to the ethical and juridical norms of scientific researches in biology, medicine and related areas corresponding to laws of Russian Federation and international GLP standards (Good Laboratory Practice). All efforts were made to minimize animal suffering. Rats were deeply anesthetized using excessive Zoletil (30 mg/kg) and sacrificed by decapitation. Anesthesia and euthanasia were performed by the trained staff member who was approved and the licensed by the Bioethics Committee of M.V. Lomonosov Moscow State University. The specimen of mitochondria from rat liver with protein concentration of 50 mg/ml was placed in a hypotonic solution containing 0.01 M sucrose and 15 mM KCl, incubated in ice for 10 min under stirring, and centrifuged at 20 000g for 15 min. The precipitate was resuspended in a small volume of a 0.25 M solution of sucrose on a homogenizer with Teflon pistil, placed in a hypertonic solution containing 150 mM KCl, incubated in ice for 10 min under stirring, and centrifuged at 20 000g for 15 min. The prepared mitoplasts without cytochrome *c* were resuspended in 0.25 M sucrose solution; its aliquots were frozen in liquid nitrogen and stored at −70°C.

### Measurement of the succinate-cytochrome *c* reductase activity

The succinate-cytochrome *c* reductase activity of mitoplasts was measured on a spectrophotometer at 550 nm and 30°C [28]. A sample (2 ml) contained the incubation medium (0.15 M sucrose, 20 mM KCl, 20 mM Tris-HCl, pH 7.4, 5 mM NaN_3_), 20μl of the specimen of mitoplasts from rat liver (10 μg of the protein per ml), and oxidized cytochrome *c*. The reaction was initiated by adding potassium succinate to a final concentration of 10 mM. The activity was expressed in μmol of the reduced cytochrome *c* for 1 min per mg of the mitoplast protein. The standard measurement error was no higher than 10% in all cases.

### Measurement of the cytochrome *c* oxidase activity of the mitoplasts

The cytochrome *c* oxidase activity of the mitoplasts was measured with an ammeter using a closed platinum electrode at 20°C [29]. A sample (1.3 ml) contained the incubation medium (0.15 M sucrose, 20 mM KCl, 20 mM Tris-HCl, pH 7.4, 10 mM ascorbic acid), 15 μl of the mitoplast specimen (65 μg of the protein per ml), and oxidized cytochrome *c*. The reaction was initiated by adding TMPD to a final concentration of 0.2 mM. The activity was expressed in μg atoms of the adsorbed oxygen for 1 min per mg of the mitoplast protein. The standard measurement error was no higher than 15% in all cases.

### Calculation of the kinetic parameters of the reactions

The enzymatic reactions (succinate:cytochrome *c* reductase and cytochrome *c* oxidase) were considered to correspond to Michaelis–Menten kinetics during the measurements. The graph of the dependence of the reaction rate on the substrate concentration was plotted using the Origin 7.0 software program (Microcal, United States) in Lineweaver–Burk double opposite coordinates (1/*A*, 1/S)

$$\frac{1}{A}=\frac{Km}{Amax}\cdot \frac{1}{{\left[S\right]}_{0}}+\frac{1}{Amax}$$

where *K*m is the Michaelis constant, [S]_0_ is the initial concentration of the substrate in the reaction mixture, *A* and *A*max are the measured and the maximum **reaction rates**, respectively. The kinetic parameters of the reactions (*K*m and *A*max) were calculated from this equation.

### Analytical methods

All stages of isolation and purification of the proteins were controlled by electrophoresis in 12% Tris-tricine PAAG under denaturing conditions in the presence of 1% SDS [30]. Concentrations of the prepared mutant proteins were determined on a spectrophotometer at 409 nm taking into account the molar absorption coefficient of the oxidized cytochrome *c* (1.06×10^5^M^-1^ cm^-1^) [31]. The quantitative content of the total protein in the mitoplast specimens was evaluated using the biuret reaction [32].

### Resonance Raman and surface-enhanced Raman spectroscopy of cytochrome *c*

The RRS and SERS spectra of wild-type and mutant cytochrome *c* molecules were recorded using an InVia Raman microspectroscope (Renishaw, UK) with the special Macrokit Renishaw holder, 532 nm laser, a lens with NA 0.02. Laser power for the recording RRS and SERS spectra was 3 and 0.3 mW, respectively. The spectrum accumulation time was 20 s. All measurements were performed in 10 mM NaPi buffer, pH 7.0, 22°C. Silver (Ag) colloid was prepared as described in [33], variant C. Briefly, Ag colloid was obtained by reducing AgNO_3_ with hydroxylamine hydrochloride under basic conditions. In order to record the SERS spectra, the cytochrome *c* solution (10^−6^ M) was mixed with Ag colloid at a 3:2 volumetric ratio. The SERS spectra were recorded immediately after mixing. In RRS experiments, we used cytochrome *c* solutions at the concentration of 1 mM. Cytochrome *c* reduced with sodium dithionite was added into the experimental probe 2–3 min before spectrum recording. In all cases, the number of independent measurements was 3–4.

## Results and discussion

### Formulation of hypothesis

**Fig 1:**
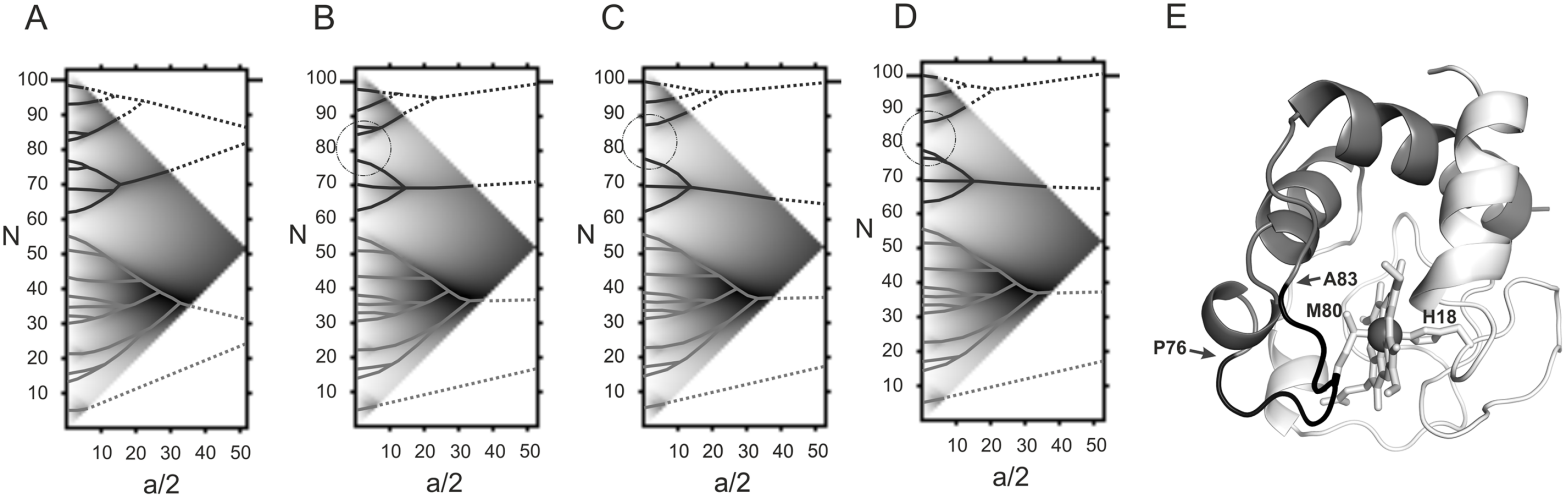
The informational structure of horse cytochrome *c* and its mutant forms: Result of the analyzis of the amino acid sequence using the ANIS method. (A) The hierarchically organized highest rank ELIS (continuous lines) and the fragments of the bipartite graph that cannot be revealed using the ANIS method (dashed line). X axis is the size of the smoothing interval a/2 [21], Y axis is the number N of amino acid in the primary structure of horse cytochrome *c*; (B), (C), (D) The hierarchically organized highest rank ELIS in mutant forms T78S/K79P, I81Y/A83Y/G84N, T78N/K79Y/M80I/I81M/F82N, respectively; (E) The spatial structure of horse cytochrome *c* (1HRC.PDB). The highest rank ELIS in the spatial structure of cytochrome c are shown. His18 and Met 80 residues coordinated to the Fe atom are indicated. The ADD- site (P76-A83) with the abnormally low density of first rank ELIS is shown by the arrows.

The amino acid sequence of horse cytochrome *c* was analyzed using the ANIS method [20]. According to the calculations, the informational structure of cytochrome *c* is represented by a bipartite graph, i.e. it consists of two independent hierarchically organized top-ranking ELIS [18]. The ELIS formed by the *N*-terminal portion of the primary protein structure comprises residues 1–58, while the ELIS formed by the *C*-terminal portion contains residues 59–104 (Fig 1A). It should be mentioned that amino acid residues His18 and Met80 that form the coordination bonds with the Fe atom reside in ELIS of different rank (Fig 1E). Another heme-containing protein, the β-subunit of hemoglobin, have a similar organization of the informational structure, i.e. its amino acid residues coordinated to the Fe atom also belong to different top-ranking ELIS [19]. According to XRD data, the oxidized and reduced forms of the β-subunit of hemoglobin differ in terms of heme conformation and position of the Fe atom with respect to the plane of the porphyrin ring [16]. We suggested that the mechanisms of functioning of the β-subunit of hemoglobin and horse cytochrome *c* are similar. They are related to the determinate mobility of the spatial structure fragments corresponding to the top-ranking ELIS due to which heme conformation is changed and the Fe atom is displaced out of the plane of porphyrin ring.

In addition to detecting hierarchically organized elements in amino acid sequences, the ANIS method allows one to study the local density of arrangement of elements of the bottom hierarchical level of the informational structure (first-rank ELIS). The low density of the first-rank ELIS in ADD- sites characterizes their high ability to undergo conformational rearrangements (flexibility) [20].

It was demonstrated that both cytochrome *c* and the β-subunit of hemoglobin have only one ADD- site. In both proteins, this site resides between the top-ranking ELIS and hosts one of the two amino acid residues coordinated to the Fe atom. In the informational structure of cytochrome *c*, the ADD- site contains residues 76–83 (Fig 1E). Determinate mobility of the top-ranking ELIS (Fig 1E) with respect to each other can be implemented as conformational rearrangements on the only ADD- site. We believe that this changes the whole heme conformation and the interaction between the Met80 residue and the Fe atom, thus ensuring functioning of cytochrome *c*. The experimental data support the structural lability of the P^76^GTKMIFA^83^ fragment of the polypeptide chain of cytochrome *c* [34, 35].

Hence, comparison of the results on the structures of cytochrome *c* and the β-chain of hemoglobin using ANIS method made it possible to formulate the hypothesis about the mechanisms of cytochrome *c* function. We suggest that heme conformational changes and Fe atom displacement out of the plane of porphyrin ring can ensure transfer of an electron from complex III to cytochrome *c* and then to complex IV. The P^76^GTKMIFA^83^ sequence plays a key role in conformational rearrangements of the heme.

To verify this hypothesis we designed the mutant variants of cytochrome *c*, where amino acid substitutions were introduced in the P^76^GTKMIFA^83^ fragment and reduced its conformational mobility. It was assumed that the introduced amino acid substitutions convert the only ADD- site into the ADD+ site characterized by an abnormally high density of the first rank ELIS [18, 19]. This modification of the fragment should reduce the ability of heme porphyrin to undergo conformational rearrangements required for efficient interaction with electron donor and acceptor and, therefore, to decrease the electron transport activity of the mutant variants of cytochrome *c*.

Designing amino acid substitutions in the polypeptide chain of cytochrome *c* that would increase the density of first-rank ELIS in 76–83 region and, therefore, would reduce the conformational mobility of this fragment was not a simple task. The challenges are related to the fact that the regularities of arrangement of amino acid residues giving rise to first-rank ELIS have not been studied yet. However, while analyzing the informational structures of proteins in the entire *E*.*coli* proteome, the frequency of amino acid residues in first-rank ELIS was found to decrease in the series: G>A> V> L>S> E> I> R> T> K> D> P> F> N> Q> Y> H>C>M>W. These data were used as a basis for designing the mutant variants of cytochrome *c*. The following eight variants of amino acid substitutions for the sequence 76–83 of cytochrome *c* were proposed: K79V/I81L/F82R, T78A/K79A/I81A/F82T/T89A, I81L/F82S/A83S/G84A, I75G/G77R/T78I, T78A/K79A/I81A/F82T, I81Y/A83Y/G84N, T78N/K79Y/M80I/I81M/F82N, and T78S/K79P. Fig 1B–1D show the changes in the architecture of the highest rank ELIS in the site 76–83 of the mutant forms of cytochrome *c*.

The mutant genes of eight variants of cytochrome *c* were obtained within the previously modified plasmid vector pBP(CYCS) [23] for coexpression of the genes of horse cytochrome *c* and heme ligase from yeast cells [24]. The corresponding recombinant proteins were produced in JM-109 *E*.*coli* cells in the absence of the expression inducer [24]. We should note that expression of certain mutant genes was unstable and the yield of the corresponding target proteins decreased 3-5-fold compared to that for non-modified cytochrome. The target proteins were purified using the combination of cation exchange and adsorption chromatography [25, 26].

### The activities of mitoplasts in the presence of mutant cytochromes *c*

The ability of the mutant variants of cytochrome *c* to interact with ubiquinol–cytochrome *c* reductase (complex III) and cytochrome *c* oxidase (complex IV) was studied in the system of rat liver mitoplasts containing complexes III and IV and being deficient in endogenous cytochrome *c* [28].

**Fig 2:**
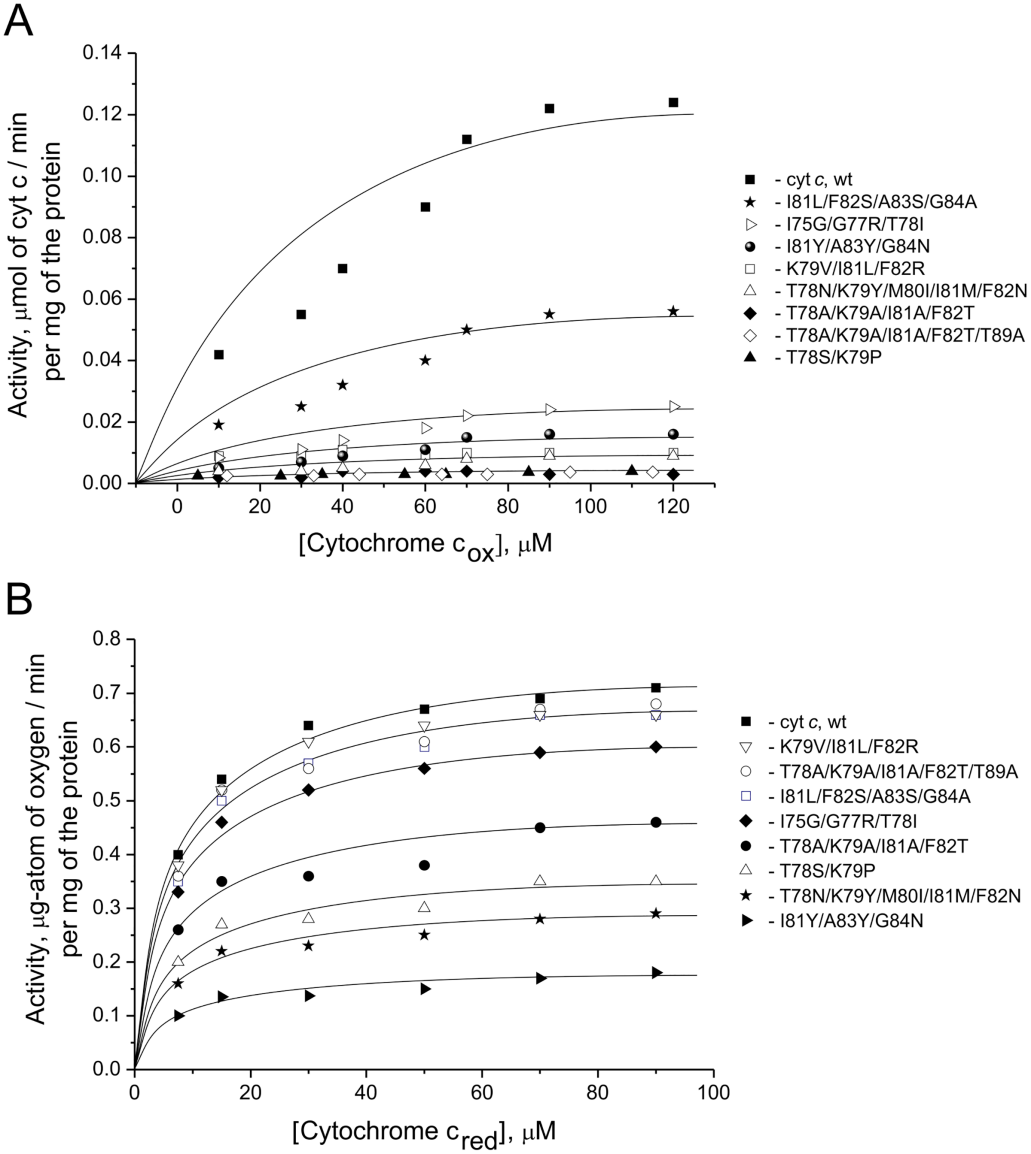
Activities of succinate-cytochrome *c* reductase and cytochrome *c* oxidase. The succinate–cytochrome *c* reductase (A) and cytochrome *c* oxidase (B) activity of rat liver mitoplasts without cytochrome *c* as a function of concentrations of the added horse heart cytochrome *c*. Residual activity for the mutant forms of cytochrome *c* evaluated in percents from wild-type cytochrome *c* activity (in brackets, after mutant labeling).

The succinate–cytochrome *c* reductase activity was measured spectrophotometrically at 550 nm and based on reduction of the completely oxidized exogenous cytochrome *c* [29]. In this reaction, potassium succinate donated electrons to the respiratory chain to complex II (succinate dehydrogenase) and further to ubiquinone, complex III, and cytochrome *c*. In order to prevent electron transfer from cytochrome *c* to cytochrome *c* oxidase, complex IV was inhibited by adding sodium azide. The succinate–cytochrome *c* reductase activity in mitoplasts measured in the presence of mutant variants of cytochrome *c* was significantly reduced by at least 45% of the activity level in the presence of wild-type cytochrome *c* (Fig 2A, Table 2). The greatest decrease in mitoplast activity (by 97% of that in the presence of wild-type cytochrome *c*) was observed when the T78A/K79A/I81A/F82T, T78A/K79A/I81A/F82T/T89A, and T78S/K79P mutant variants were added to the medium. We should mention that the residual succinate–cytochrome *c* reductase activity in the presence of these proteins is comparable to mitoplast activity in the presence of the previously obtained cytochrome *c* variants with six and eight residue substitutions that were subsequently used for detecting superoxide anion radical [11].

**Table 2:** Comparison of the kinetic parameters of the succinate:cytochrome *c* reductase and cytochrome *c* oxidase reactions in rat liver mitoplasts in the presence of the externally added WT or mutant cytochromes *c*. The ratios of the maximum reaction rates in the presence of mutant forms of cytochrome *c* to the reaction rate in the presence of a WT protein *A*max(mut)/*A*max(wt) are also calculated.

Mutant cytochrome *c*	Succinate:cytochrome *c* reductase reaction			Cytochrome *c* oxidase reaction		
	*A*max, μmol of the cyt *c* /min × mg of the protein	*A*max(mut)/ *A*max(wt), %	*K*m, μM of cyt *c*	*A*max, μg atom of the oxygen/min per 1 mg of the protein	*A*max (mut)/ *A*max (wt), %	*K*m, μM of cyt *c*
**cyt *c*, wt**	0,123	100	20,93	0,733	100	5,40
**I81L/F82S/A83S/G84A**	0,055	44,7	20,73	0,675	92,1	5,77
**I75G/G77R/T78I**	0,024	19,5	20,73	0,636	86,7	6,47
**I81Y/A83Y/G84N**	0,016	13,0	24,70	0,173	23,6	5,00
**K79V/I81L/F82R**	0,010	8,1	1,24	0,717	97,8	6,19
**T78N/K79Y/M80I/I81M/F82N**	0,008	6,5	10,96	0,283	38,6	4,96
**T78A/K79A/I81A/F82T**	0,004	3,2	8,94	0,453	61,8	5,31
**T78A/K79A/I81A/F82T/T89A**	0,003	2,4	5,08	0,677	92,3	5,31
**T78S/K79P**	0,004	3,2	17,37	0,348	47,5	5,04

The cytochrome *c* oxidase activity was measured amperometrically using a closed platinum electrode [36]. The reaction was initiated by adding ascorbic acid to the reaction medium. The ascorbic acid acted as an electron donor for tetramethyl-*p*-phenylene diamine (TMPD), which, in its turn, transferred electrons to oxidized cytochrome *c*. The reduced cytochrome *c* then transferred electrons to complex IV that reduced oxygen to water. We monitored the activity of cytochrome *c* oxidase relatively to the decrease in oxygen concentration in the reaction mixture. The cytochrome *c* oxidase activity of rat liver mitoplasts was reduced to 86, 62, and 47% of that of wild-type cytochrome *c* in the presence of I75G/G77R/T78I, T78A/K79A/I81A/F82T, and T78S/K79P variants, respectively (Fig 2B, Table 2). A significant decrease to 38% of the mitoplast activity measured in the presence of wild-type cytochrome *c*, was observed for the variant with T78N/K79Y/M80I/I81M/F82N substitutions. The maximum reduction of mitoplast activity (to 24% of the initial activity) was observed in the presence of the I81Y/A83Y/G84N mutant variant. Adding the variants carrying the K79V/I81L/F82R, I81L/F82S/A83S/G84A, and T78A/K79A/I81A/F82T/T89A mutations to the medium almost did not change the cytochrome *c* oxidase activity of mitoplasts.

Studies of the interaction between the mutant variants of cytochrome *c* and the respiratory chain complexes showed that their ability to exchange an electron with complexes III and IV of the respiratory chain in the mitoplast system decreased significantly. The maximum decreasein the succinate–cytochrome *c* reductase activity (to ~3% of the maximal reaction rate of WT cytochrome *c*) was observed for three mutant variants (T78A/K79A/I81A/F82T, T78A/K79A/I81A/F82T/T89A, and T78S/K79P) of cytochrome *c*. Simultaneous reduction of cytochrome *c* oxidase activity of mitoplasts (to ~47% of the activity in the presence of wild-type cytochrome *c*) was indicated only for one of them. The greatest decrease in cytochrome *c* oxidase activity (to ~24% of the initial level) was observed for the variant containing the I81Y/A83Y/G84N substitutions, which was also characterized by significant decrease in the succinate:cytochrome *c* reductase activity of mitoplasts (to 13% of the maximum reaction rate of the WT cytochrome *c*). Furthermore, the mutant variant T78N/K79Y/M80I/I81M/F82N exhibited a considerably lower ability to interact both with complex III and with complex IV (~7 and 39% of the WT levels, respectively).

It should be noted that Km values of the cytochrome **c** oxidase reaction did not show significant changes for all tested mutant variants of cytochrome c. With this, we suggest that the significant decrease of Amax values in the reaction of the oxidation of cytochrome c mutants is due to the change in the rate of the electron transfer along electron carriers. At the same time, the decrease of Km values of the succinate-cytochrome *c* reductase reaction was significant for the mutant cytochromes T78N/K79Y/M80I/I81M/F82N,T78A/K79A/I81A/F82T, T78A/K79A/I81A/F82T/T89A, K79V/I81L/F82R (about 2, 2, 4 and 17 times decrease, respectively). Such decrease may indicate that the observed subside in the reaction rate of the reduction of these mutants is caused by worsening their ability to form active complexes with the corresponding redox partner in complex III.

### Resonance Raman and surface-enhanced Raman spectroscopy of mutant cytochromes *c*

Our next step was to study the conformational changes in the heme of those cytochrome *c* mutant forms (I81Y/A83Y/G84N, T78N/K79Y/M80I/I81M/F82N, T78S/K79P) that demonstrated the most pronounced decrease in the activity of the electron transport chain. For this purpose, we employed resonance Raman and surface-enhanced Raman spectroscopy [33, 37–41]. The characteristic feature of cytochrome *c* is that Raman scattering of its oxidized form is much less intensive than that of the reduced form [39–41]. In addition, it turned out that Raman scattering intensity of all studied reduced mutant cytochrome *c* forms was at least two times lower than that of the WT form. Raman scattering of the oxidized mutants was below the detection limit of the spectrometer and therefore it was impossible to record their resonance Raman spectra. Therefore, we used SERS with silver colloid to compare heme conformations in oxidized mutants and WT cytochrome *c* molecules.

#### Oxidized cytochromes

**Fig 3:**
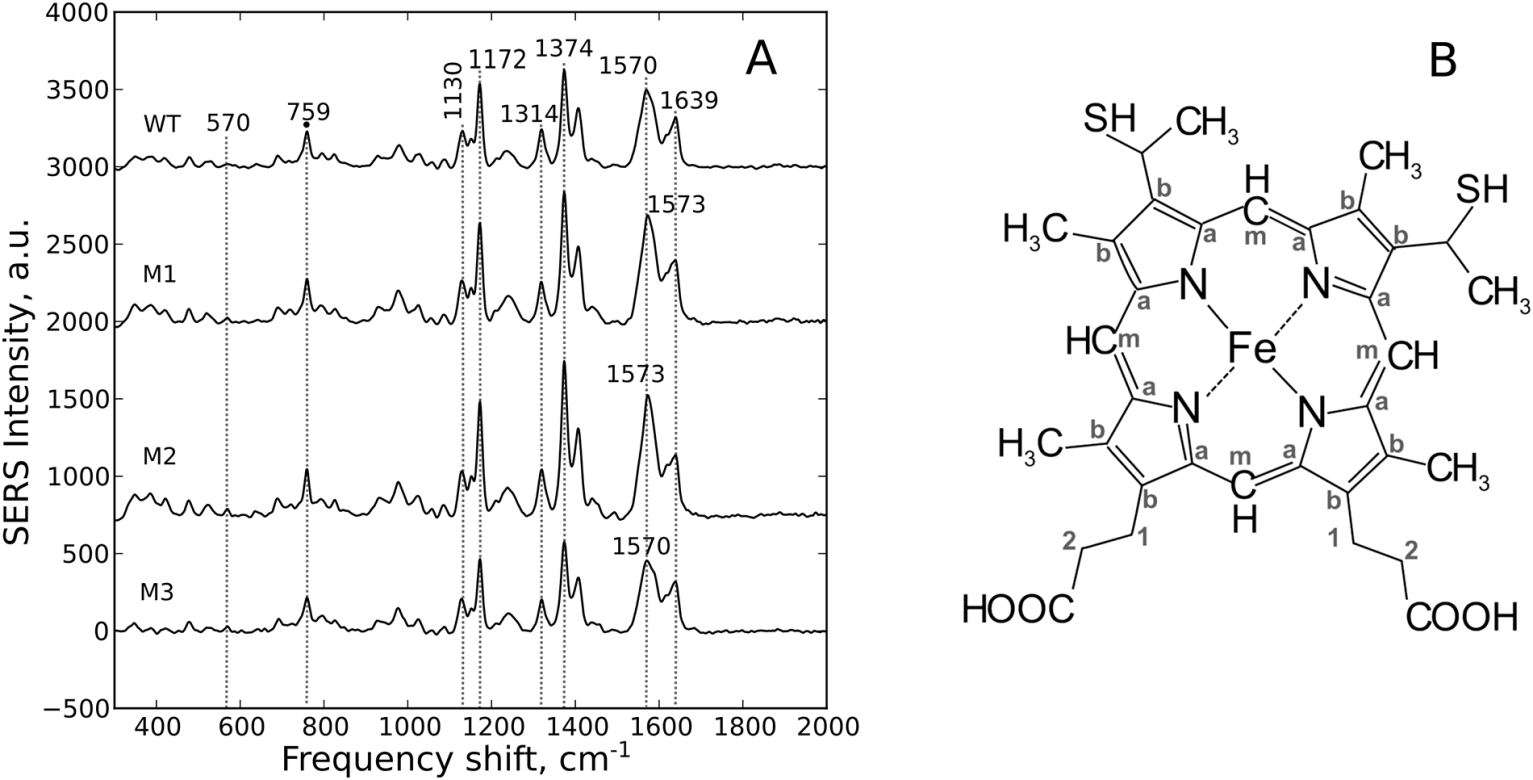
SERS study of oxidized cytochromes. **(A)** The SERS spectra of the studied cytochromes in the oxidized state: WT—wild type; M1—T78N/K79Y/M80I/I81M/F82N, M2—T78S/K79P, M3—I81Y/A83Y/G84N. For clearer presentation, the spectra are shifted in vertical position. Numbers above peaks indicate positions of their maxima used in the analysis. X axis is a frequency shift, cm^-1^; Y axis is SERS intensity, a.u. (B) Structural formula of heme *c* with numeration of C atoms.

The SERS spectra of oxidized WT and mutant cytochrome c molecules contain typical peaks of the SERS spectra of heme-containing proteins [37, 38, 42] (Fig 3A). Thus, the SERS spectra of all studied oxidized WT and mutant oxidized cytochromes mixed with Ag colloid demonstrate a set of intensive peaks corresponding to heme molecules of cytochromes with the position of their main maxima around 750, 1130, 1172, 1314, 1374, 1570–1573 and 1639 cm^-1^ (Fig 3A). These peaks originate from the normal group vibrations of pyrrole rings (bonds C_a_N, C_a_NC_a_, C_a_C_b_; peaks at 750, 1172, 1374 cm^-1^), methine bridges (bonds C_a_C_m_, C_a_C_m_H, peaks at 1570–1573, 1639 cm^-1^), side radicals in the heme molecule (C-CH_3_, 1130 cm^-1^) and all heme bonds (1314 cm^-1^) (Fig 3B). All SERS spectra contain the peak with the maximum position at 1314 cm^-1^ that is a characteristic feature of heme *c*, but not *b* [37, 39, 41, 42]. There are also other less intensive peaks that we did not use in the analysis except for the peak with the maximum position at 570 cm^-1^ (known as ν21 mode) (Fig 3A). This mode is a marker of the heme ruffling deformation [17, 43, 44]. Multiple studies on isolated cytochrome *c* and cytochrome *c* in mitochondria demonstrate that the intensity of the ν21 mode changes roughly proportional to the degree of the heme ruffling deformation [17, 43–46]. Sun and co-authors proposed to use the ratio of the peak intensities at 570 and 1374 cm^-1^ (I_570_/I_1374_) as a measure of the ruffling deformation of the heme *c* molecule. The ruffling diformation of heme involves a pyrrole-ring twisting about the Fe–N bond and is the predominant out-of-plane distortion found in *c*-type cytochromes [47, 48]. The degree of the heme ruffling distortion affects cytochrome *c* function. Thus, NMR experiments and the density functional theory computation show that the the electron transfer rate to the ferric heme decreases as a function of the ruffling deformation [49].

**Fig 4:**
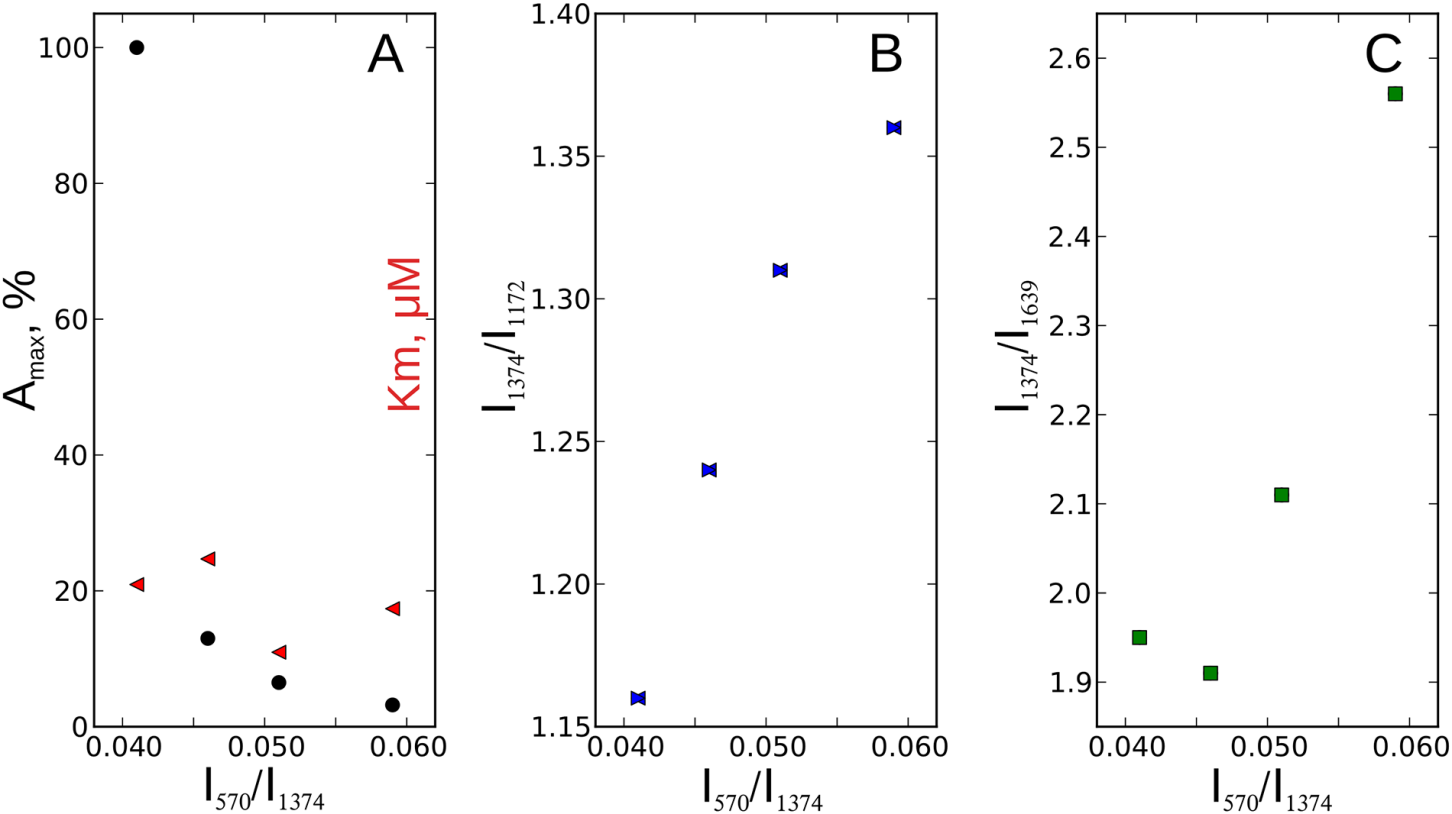
The relations between SERS data, activity and Km value of succinate:cytochrome c reductase. A: the relation between I_570_/I_1374_ ratio and the activity and Km value of the succinate:cytochrome c reductase (black circles and red triangles, respectively). The activity of the succinate: cytochrome c reductase is shown in % from the activity value in WT cytochrome c. B and C: the relations between I_570_/I_1374_ and I_1374_/I_1172_ ratios and I_570_/I_1374_ and I_1374_/I_1639_ ratios, respectively.

We found, that the peak at 570 cm^-1^ is more pronounced in mutants than in WT cytochrome c and that I_570_/I_1374_ ratio is significantly higher in mutants T78N/K79Y/M80I/I81M/F82N and T78S/K79P, than in WT cytochrome c (Table 3). This indicates that oxidized mutants T78N/K79Y/M80I/I81M/F82N and T78S/K79P have higher degree of the ruffling out-of-plane heme distortion, than WT cytochrome c. We also demonstrated, that the I_570_/I_1374_ ratio increases in a raw WT—I81Y/A83Y/G84N—T78N/K79Y/M80I/I81M/F82N—T78S/K79P while the maximal rate of the cytochrome *c* reduction decreases (Fig 4A). We did not observe any correlation between the I_570_/I_1374_ ratio and the Km value for the succinate:cytochrome c reductase (Fig 4A). Based on these findings as well as on literature data on the heme *c* ruffling distortion [49] we suggest, that mutants T78N/K79Y/M80I/I81M/F82N and T78S/K79P have slower electron transport rate, than WT cytochrome *c*.

**Table 3:** The intensity ratios of selected peaks in the SERS spectra of oxidized wild-type cytochrome *c* and its mutants. Data are shown as mean values ± SE. *p<0.05 compared to wild-type cytochrome *c* (the non-parametric Kruskal-Wallis test with Dunn's multiple comparison test).

Protein	I_572_/I_1374_	I_1374_/I_1172_	I_1130_/I_1172_	I_1374_/I_1639_
**WT**	0.041±0.005	1.16±0.12	0.43±0.025	1.95±0.02
**Mutant 1** T78N/K79Y/M80I/I81M/F82N	0.051±0.002*	1.31±0.11	0.41±0.045	2.11±0.02*
**Mutant 2** T78S/K79P	0.059±0.003*	1.36±0.11*	0.39±0.02*	2.56±0.03*
**Mutant 3** I81Y/A83Y/G84N	0.046±0.016	1.24±0.09	0.45±0.04	1.91±0.03

To characterize in-plane conformational changes in heme molecules of cytochrome *c* mutants we analyzed relative intensities of SERS peaks at 1130, 1172, 1374 and 1639 cm^-1^. We observed in-plane conformational changes in heme molecules of T78N/K79Y/M80I/I81M/F82N and T78S/K79P mutants that were manifested as an increase in the ratio between the peak intensities at 1374 and 1639 cm^-1^ (I_1374_/I_1639_) (Table 1). This change corresponds to the possible obstruction of the vibrations of methine bridges with respect to the symmetric vibration of pyrrole rings. Besides, one of the peaks corresponding to methine bridge vibrations (at 1573 cm^-1^) in the SERS spectra of oxidized mutants T78N/K79Y/M80I/I81M/F82N and T78S/K79P is shifted to the higher frequency range as compared to WT cytochrome *c* and mutant 81Y/A83Y/G84N (Fig 3). This provides an evidence that methine bonds are shorter in T78N/K79Y/M80I/I81M/F82N and T78S/K79P mutants than in WT cytochrome *c* and in I81Y/A83Y/G84N mutant [45, 50]. Furthermore, we observed the following features in the SERS spectra of T78S/K79P mutant: (i) the significant increase in the I_1374_/I_1172_ ratio demonstrating that asymmetric vibrations of pyrrole rings are less pronounced compared to the symmetric vibrations of pyrrol rings and (ii) the decrease in the intensity ratio I_1130_/I_1172_, corresponding to the obstruction of vibration of the C_b_-CH_3_ bond compared to asymmetric vibrations of pyrrole rings. The observed modifications in the SERS spectra of oxidized T78S/K79P mutant indicate that pyrrole rings change their in-plane mobility while C-CH_3_ side radicals and methine bridges between pyrroles are less mobile, than in the heme of WT cytochrome c. Changes in SERS spectra of T78N/K79Y/M80I/I81M/F82N mutant also indicate the lower mobility of its porphyrin comparing to the WT cytochrome c. Importantly, we observed almost linear correlation between the ratios I_1374_/I_1172_, I_1374_/I_1639_ and I_570_/I_1374_. meaning that the heme ruffling changes in the parallel to the changes in the vibrations of pyrrole rings and methine bridges. Thus, the increase in the degree of the ruffling distortion in the raw WT—I81Y/A83Y/G84N—T78N/K79Y/M80I/I81M/F82N—T78S/K79P is accompanied by the deterioration of the assymetric vibrations of pyrrole rings and stretching of the methine bridges (Fig 4B and 4C).

#### Reduced cytochromes

**Fig 5:**
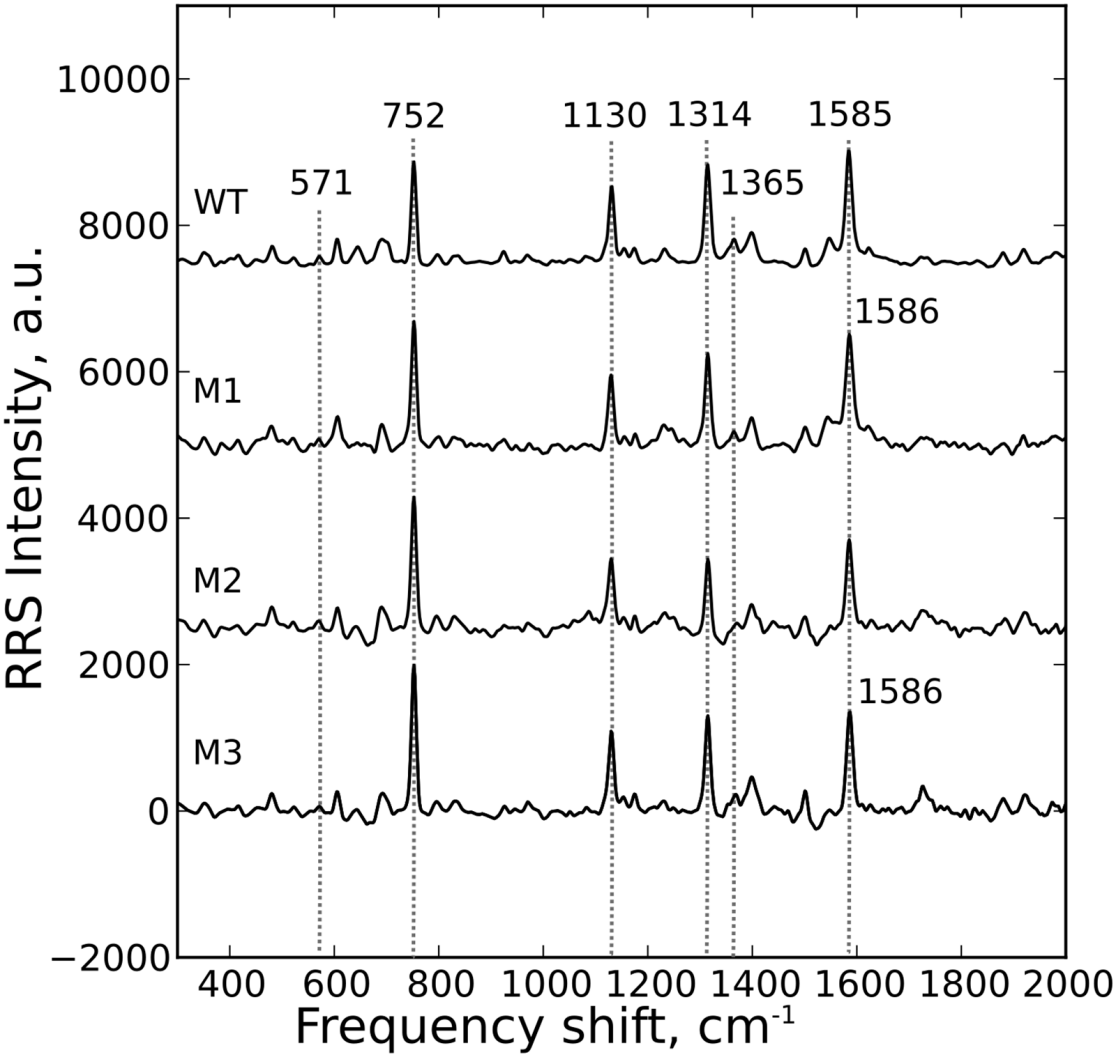
Resonance Raman study of reduced cytochromes. The RRS spectra of the studied cytochromes in the reduced state: WT—wild type; M1—T78N/K79Y/M80I/I81M/F82N, M2—T78S/K79P, M3—I81Y/A83Y/G84N. For clearer presentation, the spectra are shifted in vertical position. X axis is a frequency shift, cm^-1^ and Y axis is RRS intensity, a.u.

In the resonance Raman spectra (RRS) of all the reduced cytochrome *c* molecules we observed four intensive peaks: 752 (symmetric vibrations of pyrrole rings), 1130 (vibrations of C_b_-CH_3_ side radicals), 1314 (vibrations of all heme bonds), and 1585–1586 cm^-1^ (vibrations of methine bridges (C_a_C_m_, C_a_C_m_H bonds) and the C_a_C_b_ bond) (Fig 5). There are also a number of other peaks with lower intensities that we did not used for the study except for the peaks with the maximum positions around 571 and 1365 cm^-1^. The peak at 1365 cm^-1^ is the same mode (ν4) as the peak at 1374 cm^-1^ in SERS spectra of oxidized cytochrome originating from the symmetric vibrations of pyrrol rings. Its shift to the shorter wavenumber range is well-known for heme-containing molecules like cytochrome *c*, hemoglobin or myoglobin [17, 41, 42, 46]. We used the ratio I_571_/I_1365_ as a measure of the ruffling deformation of heme molecule in reduced WT and mutant cytochromes [17]. We should note that SERS and RRS spectra of cytochrome *c* differ from each other and that the SERS spectra have more peaks than the RRS spectra of cytochrome *c*. This is partly due to the fact that the RRS spectra of oxidized and reduced cytochrome *c* also differ in terms of number of peaks and relative peak intensities. A slight shift in the position of the peak maxima in the SERS and RRS spectra of cytochromes is not surprising since it has already been reported for various types of biomacromolecules (including heme-containing hemoglobin [37]) and simple organic molecules [51].

Importantly, we have found that in all studied reduced mutant cytochromes the ratio I_571_/I_1365_ was significantly higher, than in WT cytochrome c (Table 4). This finding indicates that the degree of ruffling deformation of heme is higher in mutants, than in WT cytochrome *c*. However, we did not observe such a “smooth” decrease in the of Amax value with the increase in the I_571_/I_1365_ ratio as we saw for oxidized cytochromes. We also did not find a straight relation between I_571_/I_1365_ and other ratios of Raman intensities. This can indicate the absence of the direct relation of the degree of heme ruffling deformation with in-plane heme vibrations in reduced cytochromes.

**Table 4:** The intensity ratios of selected peaks in the RRS spectra of the reduced wild-type cytochrome *c* and its mutants. Data are shown as mean values ± SE. *p<0.05 compared to wild-type cytochrome *c* (non-parametric Kruskal-Wallis test with Dunn's multiple comparison test).

Protein	I_571_/I_1365_	I_752_/I_1130_	I_752_/I_1314_	I_752_/I_1585_
**WT**	0.23±0.02	1.32±0.12	1.03±0.09	0.90±0.09
**Mutant 1** T78N/K79Y/M80I/I81M/F82N	0.56±0.09*	1.76±0.06*	1.35±0.16*	1.12±0.03
**Mutant 2** T78S/K79P	0.91±0.02*	1.89±0.07*	1.90±0.06*	1.48±0.07*
**Mutant 3** I81Y/A83Y/G84N	0.34±0.05*	1.82±0.32*	1.53±0.16*	1.47±0.29

We observed that in the RRS spectra of reduced mutants T78N/K79Y/M80I/I81M/F82N and I81Y/A83Y/G84N, the peak corresponding to the vibrations of methine bridges and C_a_C_b_ bond is slightly shifted to higher frequency range compared to wild-type cytochrome *c* and T78S/K79P mutant (Fig 5). This provides an evidence that the heme ring of reduced mutants T78N/K79Y/M80I/I81M/F82N and I81Y/A83Y/G84 is more compact than that in the WT cytochrome *c* and T78S/K79P mutant [45, 50].

We also observed changes in the relative contribution of various peaks into the overall RRS spectra of reduced mutant cytochromes (Table 4): (i) an increase in I_752_/I_1130_ and I_752_/I_1585_ ratios for mutant cytochromes T78N/K79Y/M80I/I81M/F82N and T78S/K79P compared to WT cytochrome *c* corresponding to the decrease in the relative contribution of vibrations of C_b_-CH_3_ side radicals and methine bridges and C_a_C_b_ bonds compared to vibrations of the pyrrole ring and (ii) an increase in I_752_/I_1314_ ratio for all the mutants corresponding to the relative increase in contribution of vibrations of the pyrrole ring compared to all heme vibrations. All observed changes in the RRS spectra indicate that heme molecules in all mutants have worse mobility than the WT cytochrome *c*.

Summarizing, we demonstrated that hemes in both oxidized and reduced mutant cytochromes have higher degree of the heme ruffling deformation comparing to WT cytochrome that results in the decrease in the electron transport rate manifesting as the decrease in the Amax value. Wesuggest that changes in SERS and RRS spectra showing the worse mobility of hemes of mutant cytochromes are the result of the increase in the stiffness of protein in the surrounding heme, namely, in the P^76^GTKMIFA^83^ loop. In WT cytochrome *c*, this loop due to its flexibility ensures the conformational mobility of the heme and its ability to change its conformation in a way required for the optimal orientation of cytochrome *c* heme with respect to its electron donor (in complex III) or electron acceptor (in complex IV). The increase in stiffness of the protein loop near the heme ring induces ruffling deformation of the heme molecule and can obstruct in-plane vibrations of methine bridges between pyrrole rings, thus reducing heme mobility. This, in turn, can reduce the rate of electron transport to Fe atom due to the worse heme ability to change its conformation for the optimal orientation against the electron donor or acceptor.

## Conclusion

Cytochrome *c* is one of the most intensively studied proteins. Multiple research revealed conformational changes in its protein part under oxidation/reduction and interaction with its redox partners [52, 53]. It was also shown that the mobility of the protein loop containing Met80 differs for the reduced and oxidized cytochrome *c* [54] and that the heme conformation and Fe atom position relatively to the porphyrin ring depends on amino acid residues covalently binding the porphyrin [17]. However, there are no studies about the relation of the loop with Met80, heme conformation and functional properties of cytochrome *c*. In the present paper we described how the mobility of the P^76^GTKMIFA^83^ loop affected: (i) the conformation of heme in both redox states and (ii) influences functional properties of cytochrome *c* to accept and to donate electron. Indeed, we demonstrated that mutations in the P^76^GTKMIFA^83^ loop decreasing the loop mobility caused the decrease in cytochrome *c* ability to exchange electrons with complexes III and IV and affected heme conformational changes. Thus, mutants have higher degree of the ruffling deformation of heme molecules and worse in-plane mobility of porphyrin ring in the comparison with WT cytochrome *c*. It is already known, that the increase in the heme ruffling deformation is accompanied by the decrease in the electron transfer rate [49]. Here we suggest that the mobility of pyrrol rings and methine bridges in porphyrin rings is necessary for the optimal orientation of the heme towards the donor and the acceptor of electrons providing higher rate of the electron transfer. We should note that one can not exclude another additional reason of the diminished functional activity of cytochrome *c* mutants. Mutations in the P^76^GTKMIFA^83^ loop can affect secondary structure of cytochrome *c* resulting in its worse docking to complexes III and IV. Nevertheless, our suggestion about the importance of the heme mobility and ruffling deformation for the electron acceptance/donation is in the agreement with available data about the shift of the heme ring in its protein cleft under reduction/oxidation and with data on the dependence of the heme redox potential on its conformation [17, 46, 49, 55, 56]. Thus, our data about the impact of stiffness of the protein loops in the heme environment on vibrations and conformation of heme bonds agrees with papers by Sun et al. [17, 46]. They demonstrated the influence of the pentapeptide Cys14XXCys17His18 loop on heme conformation and its “ruffled” “out-of-plane” deformation that, in turn, affects the redox potential of heme and the efficiency of electron transfer between heme *c* and its electron donor/acceptor.

Our findings allow us to conclude that the P^76^GTKMIFA^83^ sequence of polypeptide chain of cytochrome *c* plays a crucial role in the electron transport function of the protein. The results confirm our hypothesis that the conformationally labile domain of the polypeptide chain ensures conformational changes in heme porphyrin. These changes are very likely required for the efficient heme orientation towards its electron donor (heme c1 in complex III) and acceptor (heme a3 in complex IV). These findings are of importance for understanding the mechanisms of functional activity of cytochrome *c* in the respiratory chain and formation of reactive complexes between cytochrome *c* and its redox partners. This can be used to construct cytochrome *c* variants with targeted properties. Moreover, the results provide an additional evidence of structural and functional significance of the loop domains in protein molecules [57–59].

## References

[1] Lange K, Hunte C. Crystal structure of the yeast cytochrome *bc1* complex with its bound substrate cytochrome *c*. Proc Nat Acad Sci USA. 2002; 99: 2800–2805. 10.1073/pnas.052704699 11880631 PMC122428

[2] Wang K, Zhen Y, Sadoski R, Grinnell S, Geren L, Ferguson-Miller S, et al Definition of the interaction domain for cytochrome *c* on cytochrome *c* oxidase. II. Rapid kinetic analysis of electron transfer from cytochrome *c* to Rhodobacter sphaeroides cytochrome oxidase surface mutants. J Biol Chem. 1999; 274: 38042–38050. 10608873 10.1074/jbc.274.53.38042

[3] Rieder R, Bosshard HR. Comparison of the binding sites on cytochrome *c* for cytochrome *c* oxidase, cytochrome *bc1*, and cytochrome *c1*. Differential acetylation of lysyl residues in free and complexed cytochrome *c*. J Biol Chem. 1980; 255: 4732–4739. 6246081

[4] Smith HT, Ahmed AJ, Millett F. Electrostatic interaction of cytochrome *c* with cytochrome *c1* and cytochrome oxidase. J Biol Chem. 1981; 256 (10): 4984–4990. 6262312

[5] Dopner S, Hidebrandt P, Rosell FI, Mauk A., von Walter M, Buse G, et al The structural and functional role of lysine residues in the binding domain of cytochrome *c* in the electron transfer to cytochrome *c* oxidase. Eur J Biochem. 1999; 261(2): 379–391. 10215847 10.1046/j.1432-1327.1999.00249.x

[6] Roberts VA, Pique ME. Definition of the interaction domain for cytochrome *c* on cytochrome *c* oxidase. III. Prediction of the docked complex by a complete, systematic search. J Biol Chem. 1999; 274 (53): 38051–38060. 10608874 10.1074/jbc.274.53.38051

[7] Zhang Z, Huang L, Shulmeister VM, Chi YI, Kim KK, Hung LW, et al Electron transfer by domain movement in cytochrome *bc1*. Nature. 1998; 392 (6677): 677–684. 10.1038/33612 9565029

[8] Hunte C, Solmaz S, Lange C. Electron transfer between yeast cytochrome bc(1) complex and cytochrome *c*: a structural analysis. Biochim Biophys Acta. 2002; 1555(1–3): 21–28. 12206886 10.1016/s0005-2728(02)00249-9

[9] Solmaz S.R., Hunte C. Structure of complex III with bound cytochrome *c* in reduced state and definition of a minimal core interface for electron transfer. J Biol Chem. 2008; 283 (25): 17542–17549. 10.1074/jbc.M710126200 18390544

[10] Pepelina TYu, Chertkova RV, Dolgikh DA, Kirpichnikov MP. The role of individual lysine residues of horse cytochrome c in the formation of reactive complexes with components of the respiratory chain. Bioorg Khim. 2010; 36(1):98–104. 20386582 10.1134/s1068162010010097

[11] Pepelina TY, Chertkova RV, Ostroverkhova TV, Dolgikh DA, Kirpichnikov MP, Grivennikova VG, et al Site-directed mutagenesis of cytochrome c: reactions with respiratory chain components and superoxide radical. Biochemistry (Mosc). 2009; 74(6):625–32. 19645667 10.1134/s0006297909060066

[12] Nekrasov AN. Analysis of the information structure of protein sequences: a new method for analyzing the domain organization of proteins. J Biomol Struct Dyn. 2004; 21(5):615–24. 10.1080/07391102.2004.10506952 14769054

[13] Nekrasov AN, Radchenko VV, Shuvaeva TM, Novoselov VI, Fesenko EE, Lipkin VM. The novel approach to the protein design: active truncated forms of human 1-CYS peroxiredoxin. J Biomol Struct Dyn. 2007; 24(5):455–62. 10.1080/07391102.2007.10507133 17313190

[14] Nekrasov AN, Petrovskaya LE, Toporova VA, Kryukova EA, Rodina AV, Moskaleva EY, et al Design of a novel interleukin-13 antagonist from analysis of informational structure. Biochemistry (Mosc). 2009; 74(4):399–405. 19463093 10.1134/s0006297909040075

[15] Nekrasov AN, Zinchenko AA. Hydrolases: The correlation between informational structure and the catalytic centers organization. J Biomol Struct Dyn. 2008; 25(5): 553–562. 10.1080/07391102.2008.10507202 18282010

[16] Paoli M, Liddington R, Tame J, Wilkinson A, Dodson G. Crystal structure of T state haemoglobin with oxygen bound at all four haems. J Mol Biol. 1996; 256(4):775–792. 10.1006/jmbi.1996.0124 8642597

[17] Sun Y, Benabbas A, Zeng W, Kleingardner JG, Bren KL, Champion PM. Investigations of heme distortion, low-frequency vibrational excitations, and electron transfer in cytochrome c. Proc Nat Acad Sci USA. 2014; 111 (6):6570–6575. 24753591 10.1073/pnas.1322274111 PMC4020103

[18] Ostroverkhova TV, Chertkova RV, Nekrasov AN, Dolgikh DA, Kirpichnikov MP. Design of mutant variants of horse cytochrome *c* by analysis of informational structure method and testing its biological activity. Moscow University Biological Sciences Bulletin, 2011; 66(2):65–7.

[19] Ostroverkhova TV, Chertkova RV, Nekrasov AN. Computer simulation of cytochrome c spatial structure by MD and ANIS methods In collection of scientific articles: Stochastic and computer simulation of systems and processes. Belarus, Grodno Yanka Kupala State University of Grodno; 2010 pp. 138–142.

[20] Nekrasov AN, Zinchenko AA. Structural features of the interfaces in enzyme-inhibitor complexes. J Biomol Struct Dyn. 2010; 28(1):85–96. 10.1080/07391102.2010.10507345 20476797

[21] Nekrasov AN, Anashkina AA, Zinchenko AA. A new paradigm of protein structural organization. Proceedings of the 2nd International Conference “Theoretical Approaches to BioInformation Systems” (TABIS 2013) Institute of Physics Belgrade, Serbia; 2014. pp. 1–22.

[22] QuikChange Site-Directed Mutagenesis Kit. Instruction manual. Stratagene, 1997.

[23] Pollock WB, Rosell FI, Twitchett MB, Dumont ME, Mauk AG. Bacterial expression of a mitochondrial cytochrome c. Trimethylation of lys72 in yeast iso-1-cytochrome c and the alkaline conformational transition. Biochemistry. 1998; 37(17):6124–6131. 10.1021/bi972188d 9558351

[24] Dolgikh DA, Latypov RF, Abdullaev ZKh, Kolov V, Roder H, Kirpichnikov MP. Expression of mutant horse cytochrome c genes in Escherichia coli. Bioorg Khim. 1998; 24(10):756–759. 9929736

[25] Bortolotti CA, Borsari M, Sola M, Chertkova R, Dolgikh D, Kotlyar A, et al Orientation-dependent kinetics of heterogeneous electron transfer for cytochrome c immobilized on gold: Electrochemical determination and theoretical prediction. J Phys Chem C. 2007; 111:12100–12105.

[26] Chertkova RV, Sharonov GV, Feofanov AV, Bocharova OV, Latypov RF, Chernyak BV, et al Proapoptotic activity of cytochrome c in living cells: effect of K72 substitutions and species differences. Mol Cell Biochem. 2008; 314(1–2):85–93. 10.1007/s11010-008-9768-7 18425421

[27] Johnson D, Lardy H. Isolation of Mitochondria. Meth Enzymol. 1967; 10: 94–96.

[28] Jacobs EE, Sanadi DR. The reversible removal of cytochrome c from mitochondria. J Biol Chem. 1960; 235: 531–534. 14406362

[29] Vinogradov AD, Leikin YuN, Lipskaya TYu. Mitochondrial biochemistry. Bioenergetics In Manual of practical study to animal biochemistry. Moscow, Lomonosov Moscow State University publishers 1977 pp. 19–22.

[30] Schagger H, Jagow G. Tricine-sodium dodecyl sulfatepolyacrylamide gel electrophoresis for the separation of proteins in the range from 1 to 100 kDa. Analitical biochemistry. 1987; 166: 368–379. 10.1016/0003-2697(87)90587-2 2449095

[31] Babul J, Stellwagen E. Participation of the protein ligands in the folding of cytochrome c. Biochemistry. 1972; 11(7):1195–1200. 5062485 10.1021/bi00757a013

[32] Gornal AG, Bardawill CJ, David MM. Determination of serum proteins by means of the biuret reaction. J Biol Chem. 1949; 177(2):751–766. 18110453

[33] Leopold N, Lendl B. A new method for fast preparation of highly SERS active silver colloids at room temperature by reduction of silver nitrate with hydroxylamine hydrochloride. J Phys Chem. 2003; 107:5723–5727.

[34] Hoang L, Maity H, Krishna MM, Lin Y, Englander SW. Folding units govern the cytochrome c alkaline transition. J Mol Biol. 2003; 331(1):37–43. 12875834 10.1016/s0022-2836(03)00698-3

[35] Liu W, Rumbley JN, Englander SW, Wand AJ. Fast structural dynamics in reduced and oxidized cytochrome c. Protein Sci. 2009; 18(3):670–674. 10.1002/pro.72 19241377 PMC2760373

[36] Ferguson-Miller S, Brautigan DL, Margoliash E. Correlation of the kinetics of electron transfer activity of various eukaryotic cytochromes c with binding to mitochondrial cytochrome c oxidase. J. Biol. Chem. 1976; 251:1104–1115. 2600

[37] Brazhe NA, Abdali S, Brazhe AR, Luneva OG, Bryzgalova NY, Parshina EY, et al New insight into erythrocyte through in vivo surface-enhanced Raman spectroscopy. Biophys. J. 2009; 97(12):3206–3214. 10.1016/j.bpj.2009.09.029 20006958 PMC2793362

[38] Brazhe NA, Parshina EY, Khabatova VV, Semenova AA, Brazhe AR, Yusipovich AI, et al Tuning SERS for living erythrocytes: Focus on nanoparticle size and plasmon resonance position. Journal of Raman Spectroscopy. 2013; 44(5):686–694.

[39] Ogawa M, Harada Y, Yamaoka Y, Fujita K, Yaku H., Takamatsu T. Label-free biochemical imaging of heart tissue with high-speed spontaneous Raman microscopy. Biochem Biophys Res Commun. 2009; 382 (2):370–374. 10.1016/j.bbrc.2009.03.028 19285035

[40] Brazhe NA, Treiman M, Brazhe AR, Find NL, Maksimov GV, Sosnovtseva OV. Mapping of redox state of mitochondrial cytochromes in live cardiomyocytes using Raman microspectroscopy. PLoS One. 2012; 7(9):41990. 10.1371/journal.pone.0041990 PMC3434226 22957018

[41] Brazhe NA, Treiman M, Faricelli B, Vestergaard JH, Sosnovtseva O. In situ Raman study of redox state changes of mitochondrial cytochromes in a perfused rat heart. PLoS One. 2013; 8(8):70488. 10.1371/journal.pone.0070488 PMC3757006 24009655

[42] Brazhe NA, Evlyukhin AB, Goodilin EA, Semenova AA, Novikov SM, Bozhevolnyi SI, et al Probing cytochrome c in living mitochondria with surface-enhanced Raman spectroscopy. Sci Rep. 2015; 8(5):13793. 10.1038/srep13793 PMC4561893 26346634

[43] Jordan T, Eads JC, Spiro TG. Secondary and tertiary structure of the A-state of cytochrome c from resonance Raman spectroscopy. Protein Sci. 1995; 4(4):716–728. 10.1002/pro.5560040411 7613469 PMC2143105

[44] Takahashi S, Yeh S-R, Das TK, Chan C-K, Gottfried DS, Rousseau DL. Folding of cytochrome c initiated by submillisecond mixing. Nat Struct Biol. 1997; 4(1): 44–50. 8989323 10.1038/nsb0197-44

[45] Berezhna S, Wohlrab H, Champion PM. Resonance Raman investigations of cytochrome c conformational change upon interaction with the membranes of intact and Ca2+-exposed mitochondria. Biochemistry. 2003; 42:6149–6158. 10.1021/bi027387y 12755617

[46] Sun Y, Karunakaran V, Champion PM. Investigations of the low-frequency spectral density of cytochrome c upon equilibrium unfolding. J Phys. Chem. B 2013; 117(33):9615–9625. 10.1021/jp404881k 23863217 PMC3769956

[47] Jentzen W, Ma JG, Shelnutt JA. Conservation of the conformation of the porphyrin macrocycle in hemoproteins. Biophys J. 1998; 74(2 Pt 1):753–763. 10.1016/S0006-3495(98)74000-7 9533688 PMC1302556

[48] Shelnutt JA, Song X-Z, Ma J-G, Jia S-L, Jentzen W, Medforth CJ. Nonplanar porphyrins and their significance in proteins. Chem Soc Rev. 1998; 27(1): 31–41.

[49] Liptak MD, Wen X, Bren KL. NMR and DFT investigation of heme ruffling: Functional implications for cytochrome c. J Am Chem Soc. 2010; 132(28):9753–9763. 10.1021/ja102098p 20572664 PMC2914482

[50] Adar F, Erecinska M. Spectral evidence for interactions between membrane-bound hemes: resonance Raman spectra of mitochondrial cytochrome bc1 complex as a function of redox potential. FEBS Lett. 1977; 80(1):195–200 196925 10.1016/0014-5793(77)80438-9

[51] Moskovits M, Suh JS. Surface selection rules for surface-enhanced Raman spectroscopy: calculations and application to the surface-enhanced Raman spectrum of phthalazine on silver. J Chem Phys. 1984; 88 (23):5526–5530.

[52] Scott RA, Mauk A G. Cytochrome *c*: A Multidisciplinary Approach. (Univ. Sci. Books, Mill Valley, CA). 1995.

[53] Banci L, Assfalg M. Mitochondrial cytochrome *c* In Handbook of metalloproteins, Eds. Messerschmidt A., Huber R., Weighart K., Poulos T. 2001.

[54] Volkov AN, Vanwetswinkel S, Van de Water K, Van Nuland NAJ. Redox-dependent conformational changes in eukaryotic cytochromes revealed by paramagnetic NMR spectroscopy. J Biol NMR. 2012; 52: 245–256. 10.1007/s10858-012-9607-8 22318343

[55] Takano T, Dickerson RE. Redox conformation changes in refined tuna cytochrome *c*. Proc Natl Acad Sci USA. 1980; 77(11):6371–6375. 6256733 10.1073/pnas.77.11.6371 PMC350286

[56] Ma J-G, Zhang J, Franco R, Jia S-L, Moura I, Moura JJG, et al The Structural Origin of Nonplanar Heme Distortions in Tetraheme Ferricytochromes *c*. Biochemistry. 1998; 37:12431–12442. 10.1021/bi981189i 9730815

[57] Leszczynski JF, Rose GD. Loops in globular proteins: a novel category of secondary structure. Science. 1986; 234(4778):849–855. 3775366 10.1126/science.3775366

[58] Hsiao HC, Boycheva S, Watmough NJ, Brittain T. Activation of the cytochrome *c* peroxidase of Pseudomonas aeruginosa. The role of a heme-linked protein loop: a mutagenesis study. J Inorg Biochem. 2007; 101(8):1133–1139. 10.1016/j.jinorgbio.2007.04.012 17568678

[59] Inoue T, Suzuki S, Nishio N, Yamaguchi K, Kataoka K, Tobari J, et al The significance of the flexible loop in the azurin (Az-iso2) from the obligate methylotroph Methylomonas sp. strain J. J Mol Biol. 2003; 333(1):117–124. 14516747 10.1016/j.jmb.2003.08.002

